# Symptomatic and asymptomatic secondary transmission of *Cryptosporidium parvum* following two related outbreaks in schoolchildren

**DOI:** 10.1017/S095026881400243X

**Published:** 2014-10-02

**Authors:** Ø. H. JOHANSEN, K. HANEVIK, F. THRANA, A. CARLSON, T. STACHURSKA-HAGEN, D. SKAARE, L. J. ROBERTSON

**Affiliations:** 1 Department of Microbiology, Vestfold Hospital Trust, Tønsberg, Norway; 2 Department of Clinical Science, University of Bergen, Bergen, Norway; 3 National Centre for Tropical Infectious Diseases, Department of Medicine, Haukeland University Hospital, Bergen, Norway; 4 Tønsberg Municipal Public Health Department, Tønsberg, Norway; 5 Parasitology Laboratory, Department of Food Safety and Infection Biology, Norwegian University of Life Sciences, Oslo, Norway

**Keywords:** *Cryptosporidium*, gastrointestinal infections, outbreaks, zoonoses

## Abstract

Two related outbreaks (in 2009 and 2012) of cryptosporidiosis in Norwegian schoolchildren during a stay at a remote holiday farm provided us with a natural experiment to investigate possible secondary transmission of *Cryptosporidium parvum* IIa A19G1R1. After the children had returned home, clinical data and stool samples were obtained from their household contacts. Samples were investigated for the presence of *Cryptosporidium* oocysts by immunofluorescence antibody test. We found both asymptomatic and symptomatic infections, which are likely to have been secondary transmission. Laboratory-confirmed transmission rate was 17% [4/23, 95% confidence interval (CI) 7·0–37·1] in the 2009 outbreak, and 0% (95% CI 0–16·8) in the 2012 outbreak. Using a clinical definition, the probable secondary transmission rate in the 2012 outbreak was 8% (7/83, 95% CI 4·1–16·4). These findings highlight the importance of hygienic and public health measures during outbreaks or individual cases of cryptosporidiosis. We discuss our findings in light of previous studies reporting varying secondary transmission rates of *Cryptosporidium* spp.

## INTRODUCTION

The protozoan parasite *Cryptosporidium* is, after rotavirus, the second most important cause of moderate-to-severe childhood diarrhoea in Africa south of Sahara and in South Asia [1]. In high-income countries it is an under-recognized pathogen in sporadic gastroenteritis [2–5], a leading cause of drinking-water outbreaks of gastroenteritis, and has caused several zoonotic, foodborne, and swimming pool-related outbreaks [6].

There is little published data on the secondary transmission rate of *Cryptosporidium* spp. after incidental infection or after its introduction during an epidemic. Among the published reports, very few provide species or subtype information. This is a major shortcoming, given the growing evidence for differences in ecology, pathogenicity and epidemiology of the two main human pathogenic species, *Cryptosporidium hominis* and *C. parvum*, and between different *Cryptosporidium* subtypes [7]

Secondary transmission data mainly stem from outbreak reports where case definitions are based on self-reported gastrointestinal illness and very rarely include laboratory confirmation of secondary cases. Baseline demographic data for the exposed group are often lacking and few studies have assessed the rate of asymptomatic secondary infections. A prospective cohort study in an urban slum community in Brazil, considered an endemic setting for *Cryptosporidium*, found household transmission rates of 19% [8]. The median age of the index cases was 11 months. Molecular investigations were not performed, but later studies found *C. hominis* to be the dominant species in the area [9]. Similar studies of secondary *Cryptosporidium* infection in a non-endemic, developed world setting with low HIV-prevalence have, to our knowledge, not been conducted. This could be partly due to difficulties in distinguishing between primary and secondary infections during epidemic outbreaks [10], as close contacts and index cases often have similar exposures.

Two outbreaks of cryptosporidiosis at the same recreational holiday farm, 3 years apart [11, 12] with the same subtype of *C. parvum* (GP60 allele type IIa A19G1R1) in children from the same school provided situations where this problem did not occur, as we were able to assess the rate of secondary spread to close household contacts when the children returned home, and away from the source of the initial infection. Both outbreaks occurred during organized school trips to the farm in early spring. The farm is located in a remote mountain area about 200 km from the school. There were no reported gastroenteritis cases in non-visiting students and school staff.

See Table 1 for a comparison of the key characteristics of each of the two outbreaks.

**Table 1.** Key characteristics of the two outbreaks

	2009 outbreak [12]	2012 outbreak [11]
Date of notification of outbreak	24 March	25 March
Date first human stool sample received at laboratory	20 March	24 March
Date of first laboratory-confirmed *Cryptosporidium* case	2 April	26 March
Time period cases visited the holiday farm	9 March–29 March	5 March–23 March
No. of visitors during period of visit	168	290
No. of questionnaire respondents	141	209
Questionnaire response rate	84%	72%
Male:female ratio of respondents	1:1	1:1
Age of respondents	10–14 years (73%)	10–14 years (89%)
No. of cases (clinical definition)	55	40
Risk factors found in the retrospective cohort investigations	Lack of hand washing	Physical contact with farm animals
Attack rate	39%	19%
No. of laboratory-confirmed *Cryptosporidium* cases	11	15
*Cryptosporidium* species in human samples	*Cryptosporidium parvum* (11/11)	*Cryptosporidium parvum* (15/15)
Number of human samples subtyped at the GP60 locus	10/11	4/15
GP60 allele type	IIa A19G1R1	IIa A19G1R1
Water sample analysis results	No *Cryptosporidium* oocysts detected	No *Cryptosporidium* oocysts detected
Animal samples; species tested and results	4 sheep tested: 4 *Giardia*-positive, 1 *Cryptosporidium*-positive (low oocyst numbers)	26 animals tested (goat kids, calves, horses, lambs): 4 *Cryptosporidium*-positive kids; 2 *Cryptosporidium*-positive lambs
*Cryptosporidium* species in animal samples	PCR failed to amplify	*Cryptosporidium parvum* and *C. xiaoi*
GP60 allele type	PCR failed to amplify	IIa A19G1R1

PCR, Polymerase chain reaction.

Prior to these outbreaks there had been only two documented outbreaks of cryptosporidiosis in Norway (both *C. parvum*); a small outbreak associated with calves in 2005 [13], and a hotel outbreak with 25 diarrhoeal cases in 2007 [14]. During a large waterborne giardiasis outbreak in Bergen, Norway in 2004, 115 infections with *Cryptosporidium* were identified (13 samples genotyped, all *C. parvum*), of which 22 were considered to be symptomatic [15]. In neither of these previous outbreaks nor in this other cluster of cases was any effort made to identify or investigate secondary transmission.

## MATERIALS AND METHODS

### Participants

Pupils from four different schools in eastern Norway had been at the holiday farm during week 10 (2012) or week 11 (2009 and 2012, respectively) and the study was undertaken in one of these schools. On request from the municipal public health officer the school compiled lists of all children reporting nausea, stomach pain or cramps, vomiting, fever, or other signs of acute illness. The primary caregiver in each affected household was contacted and asked to participate in a telephone interview and to submit stool samples from household members for parasite analysis. None of the household contacts had visited the holiday farm.

In the part of the study associated with the 2009 outbreak we interviewed the caregivers of all index case schoolchildren (*n* = 8) who had been on a trip to the holiday farm in week 11 (March 2009), and for whom laboratory-confirmed *Cryptosporidium* infection was subsequently detected. In the study part associated with the 2012 outbreak, we contacted the households of all schoolchildren meeting the clinical case definition (*n* = 25) who had been to the holiday farm in weeks 10 or 11 (March 2012). In 2009, all interviews were conducted on 8 April; in 2012 the interviews were conducted between 30 March and 11 April. All household members and any index children who had not already submitted a stool sample, were asked to submit one sample.

From each household member we recorded information on symptoms (presence and duration of diarrhoea, abdominal pain, vomiting, nausea, fever, other symptoms), any major chronic illnesses or compromised immunity, and relationship to the index child. Household contacts were not asked about exposures other than contact with index cases.

### Case definitions

For the purposes of this study we applied the clinical case definition used in the 2009 and 2012 outbreak investigations. A primary case (hereafter referred to as the index case) was defined as a child who had been to the holiday farm during the relevant period (see Table 1) and had experienced diarrhoea, or at least two of the following symptoms, during or within 2 weeks of returning home: vomiting, nausea, abdominal pain, fever, with a duration of symptoms >24 h. A secondary clinical case was defined as a household member of an index case, with either diarrhoea or at least two of the above symptoms, with a duration of symptoms >24 h, starting >24 h after contact with the index case, and >24 h after symptoms started in the index case. A secondary laboratory-confirmed case was defined as a household member of an index case, with detection of *Cryptosporidium* in a stool sample taken >24 h after contact with the index case, and >24 h after symptoms started in the index case.

### Stool analyses

Stool samples were submitted to the Department of Microbiology at Vestfold Hospital Trust, Tønsberg, concentrated and fixed in 4% formalin (see [11] for details) on the day of reception or the following day, before microscopy for *Cryptosporidium* oocysts by immunofluorescence antibody test (IFAT, Merifluor, Meridian Biosciences, USA). In the 2009 outbreak, all samples were anonymized (assigned a number code) and transported to the Norwegian School of Veterinary Science for parallel-blinded investigation by IFAT, for quality control purposes. Some samples were also analysed by *Cryptosporidium* polymerase chain reaction (PCR) for confirmation, genotyping and subtyping. For details of the PCR method, see [11]. Examination of stool for other pathogens was not part of the *Cryptosporidium* secondary transmission study, but was part of the initial outbreak investigation in 2009 and 2012 (for details see [11] and [12]).

### Statistical analyses

We calculated secondary transmission rates using both the clinical (2012 outbreak) and laboratory-confirmed (2009 and 2012 outbreaks) case definitions. Asymptomatic secondary infection rate was calculated. All rates were calculated by dividing the number of secondary household cases (clinical, laboratory-confirmed, asymptomatic) by the total number of exposed household contacts. We calculated 95% confidence intervals (CIs) with the statistical software program Confidence Interval Analysis (CIA) v. 2·2·0 (T. Bryant, University of Southampton, UK), using Wilson's method for single proportions [16] and Newcombe's method for comparing unpaired proportions [17]. We used two-sided Fisher's exact test for comparing unpaired proportions using Predictive Analytics Software (PASW) Statistics v. 18·0 (IBM Corporation, USA).

### Ethical considerations

Investigation of outbreaks and implementation of control measures do not require approval from an ethical review board in Norway. This is in agreement with the International Guidelines for Ethical Review of Epidemiological Studies by the Council for International Organisations of Medical Sciences (CIOMS) (1991).

## RESULTS

See Table 2 for a summary of the main findings.

**Table 2.** Secondary transmission in the 2009 and 2012 outbreaks

	2009 outbreak [12]	2012 outbreak [11]
Included primary cases; clinical case definition, *n*	Not included	24
Included primary cases; laboratory-confirmed, *n*	8	9
Primary cases confirmed by IFAT/PCR, *n*/*n*	8/8	9/6
Primary cases subtyped as IIaA19G1R1, *n*	7	4
Household contacts examined*, *n*	23	83
Clinically defined secondary cases, *n* (%)	2 (9%)	7 (8%)
Secondary cases confirmed by IFAT (%)/PCR, *n*/*n*	4 (17%)/1	0 (0%)/0
Secondary cases subtyped as IIaA19G1R1, *n*	1	0

IFAT, Immunofluorescence antibody test; PCR, polymerase chain reaction.

* All household contacts in 2012; only household contacts of laboratory-confirmed primary cases in 2009.

### Households and index cases in the 2009 outbreak

Responses were obtained from 7/8 households with laboratory-confirmed *Cryptosporidium*-positive index case children (two girls, five boys) aged 12–13 years. All eight index case specimens were genotyped as *C. parvum*, subtyping was successful for 7/8 samples, all belonged to subtype IIaA19G1R1 [11]. Median size of households was five (range 2–6). All index cases had diarrhoea; additional reported symptoms in index cases were abdominal pain (4/7), fever (2/7), and vomiting (2/7). Median duration of diarrhoea in index cases was 7 days (mean 11, range 3–28 days), with samples obtained a median of 11 days after onset of diarrhoea (mean 15, range 8–39 days), and a median of 4 days (mean 4, range −20 to 32 days) after diarrhoea resolution.

### Household contacts in the 2009 outbreak

Out of a total of 25 household contacts, 23 (12 female, 11 male) submitted stool samples, with samples obtained a median of 29 days (mean 29, range 8–39 days) after onset of diarrhoea in the index case, and a median of 20 days (mean 17, range 1–27 days) after diarrhoea resolution in the index case. Median age of household contacts was 38 years (mean 28, range 6–57 years).

### Secondary transmission in the 2009 outbreak, by laboratory-confirmed case definition

*Cryptosporidium* oocysts were detected by IFAT in samples from 4/25 contacts (all female) in three different households. In the first household *Cryptosporidium* was detected in a younger sister (age 7 years) with no symptoms, and in a mother (age 47 years) who reported diarrhoea (duration 7 days) which began 25 days after index onset of diarrhoea. In the second household, a mother (age 38 years) had *Cryptosporidium* infection but reported no symptoms. In the third household *Cryptosporidium* was detected from a mother (age 42 years) who reported diarrhoea (duration 7 days) commencing 7 days after index onset of diarrhoea. All three caregivers had close contact with the index child. The remaining household contacts did not report any symptoms. Genotyping of the sample from the adult case in the first household gave *C. parvum* subtype IIaA19G1R1. Genotyping from the index case in the same family demonstrated *C. parvum* infection but the GP60 subtype could not be determined due to poor sequence quality.

Excluding the two contacts with missing samples, a secondary transmission rate of 17% (4/23) (95% CI 7·0–37·1) was determined.

### Households and index cases in 2012 outbreak

Twenty-five households with clinical case definition index cases (13 girls and 11 boys, 12–13 years old) were contacted. One household chose not to participate in the study. Median size of the 24 included households was 4·5 persons (range 2–6).

Eighty-eight percent (21/24) of the index cases reported diarrhoea, with median duration of 5 days (mean 5·2, range 1–10 days). Additional reported symptoms were abdominal pain (88%, 21/23), fever (58%, 14/24), and vomiting (75%, 18/24). A small number of children reported headache (*n* = 2), dizziness (*n* = 1) and fatigue (*n* = 1).

Stool samples were obtained for investigation by IFAT from 18/24 index children at a median of 5 days (mean 9, range −3 to 26 days) after onset of diarrhoea and a median of 2 days (mean 4, range −8 to 25 days) after resolution of diarrhoea. Fifty percent (9/18) were positive for *Cryptosporidium* oocysts, and all the positive samples were obtained from children with diarrhoea. The IFAT-negative samples (9/18) were analysed by *Cryptosporidium* spp. PCR, also with negative results. Six of nine IFAT-positive samples were analysed by species-specific PCR and found positive for *C. parvum*. Four of these were successfully subtyped to GP60 allele type IIaA19G1R1 (see [11]).

### Household contacts in the 2012 outbreak

Out of a total of 83 household contacts, 48 (24 female, 24 male) submitted stool samples, with samples obtained a median of 23·5 days (mean 21, range 12–29 days) after the return of the index case to the household. Samples were obtained a median of 19 days (mean 16, range 3–27 days) after onset of diarrhoea in the index case, and a median of 11 days (mean 12, range 1–26 days) after diarrhoea resolution in the index case. Median age of household contacts was 38·5 years (mean 30, range 2–51 years).

### Secondary transmission in the 2012 outbreak, by clinical case definition

Out of 83 contacts in 24 households (47% female, 53% male; median age 38·5, range 2–51 years), seven met the clinical case definition (three female and four male; median age 43, range 16–47 years). They belonged to four different households, but only one of the four index cases (one associated household case) had laboratory-confirmed cryptosporidiosis. One index case and two associated household cases failed to submit stool samples. Of the remaining two index cases, with three and one associated household case, respectively, the first was negative by supplementary *Cryptosporidium* PCR; the second index case was not tested by PCR due to insufficient sample volume. Samples from these four household contacts were obtained a median of 22 days (range 21–22 days) after their own diarrhoea onset, and a median 19 days (range 19–20 days) after diarrhoea resolution. Secondary transmission rate using the clinical case definition was therefore 8·4% (7/83, 95% CI 4·1–16·4).

### Secondary transmission in the 2012 outbreak, by laboratory-confirmed case definition

*Cryptosporidium* oocysts were not detected in any of the 48 samples received from household contacts.

Nine households had a laboratory-confirmed index case, with a total of 35 contacts. We received stool samples from 19 of these, with samples obtained a median of 11 days (mean 13·4, range 3–24 days) after onset of diarrhoea in the index case. As *Cryptosporidium* oocysts were not detected in any of these samples, using the stricter laboratory-based case definition we found a secondary transmission rate of 0% (95% CI 0–16·8).

## DISCUSSION

In the 2009 outbreak, four (17%) household contacts had laboratory-confirmed *Cryptosporidium* infection. No confirmed household infections occurred in the 2012 outbreak, although seven (8%) household contacts satisfied the clinical case definition.

Reports from other outbreaks have shown varying secondary transmission rates and suggest that host factors such as age and comorbidity may impact transmission rates. Differences in secondary transmission rates between different species have not been studied systematically. During the 1993 Milwaukee waterborne outbreak of cryptosporidiosis, probably caused by *C. hominis*, the secondary transmission rate was 5% in household members of visitors to the Milwaukee area. The index cases were adults with laboratory-confirmed or clinical cryptosporidiosis [10]. Outbreak investigations in day-care centres have found considerably higher transmission rates [18–20] – reaching as high as 23% in one study [18] – indicating that the age of the index case may have a considerable influence on the risk of transmission. Secondary transmission rates of 50% have been reported in group residential homes for HIV-infected patients in the USA [21]. Our secondary transmission rate findings are similar to those reported (8–10%) in the households of children aged 9–12 years after a swimming pool outbreak with *C. parvum* in Sweden [22]. However, stool samples from secondary cases were not investigated in that study, and thus secondary transmission is based entirely on case definition from clinical symptoms. This means that not only may symptoms have had different aetiologies than *Cryptosporidium*, but also that asymptomatic secondary cases would not be identified.

Although our study provides interesting information, there are some limitations. First, there was no control group and household contacts were not asked about other exposures than contact with index cases. Little is known of the baseline incidence of *Cryptosporidium* infections in Norway and cryptosporidiosis has only been notifiable since 2012. *C. parvum* subtype IIaA19G1R1 has been occasionally reported in studies from other countries (see [23] for a recent summary) but typing is not routinely conducted in Norway. Therefore there is a small possibility that some of the four laboratory-confirmed household cases in the 2009 outbreak could have acquired *Cryptosporidium* from a different source, although this would seem unlikely. Second, data collection was conducted retrospectively and by telephone, with risk of recall bias. In addition, intermittent shedding of oocysts can occur [24], and we might have missed some cases as we only collected one stool sample from each participant. For the same reason we would not have uncovered any tertiary household infections or recurrences. Third, we could have missed some asymptomatic secondary infections in the 2012 outbreak, since 16 contacts of laboratory-confirmed index cases failed to submit samples. Assuming a similar asymptomatic secondary transmission rate as in the 2009 outbreak (9%, 95% CI 2·4–26·8) this would mean that we missed 0–4 asymptomatic infections; the best estimate is one missed asymptomatic infection.

Furthermore, due to the *ad hoc* nature of the 2009 study we only included families of laboratory-confirmed index cases. This could have introduced bias due to case ascertainment, potentially favouring inclusion of more symptomatic children. This bias was probably limited as the municipal health authority, on notification of the outbreak, recommended that all pupils with any gastrointestinal symptoms submit stool samples. In the 2012 outbreak we included and asked for stool samples from all children that met the clinical case definition in order to minimize case ascertainment bias.

We were also unable to compare secondary attack rates by the clinical case definition between the two outbreaks. However, since none of the 8% secondary cases (7/83) in the 2012 study were positive for *Cryptosporidium* by IFAT, we suspect that calculations of secondary attack rates based on self-reported diarrhoeal illness will give unreliable results.

Despite these limitations, we found that both asymptomatic and symptomatic secondary infections do occur with *C. parvum* subtype IIa A19G1R1 in developed, non-endemic settings. Laboratory-confirmed secondary transmission rate was 17% in the 2009 outbreak, and 0% in the 2012 outbreak. Although there was an absolute difference in the laboratory-confirmed secondary transmission rate of 17% compared to the 2009 outbreak, the difference was not statistically significant (95% CI −2·4 to 37·1). Using the clinical case definition, the secondary transmission rate in the 2012 outbreak was 8%. All index cases were aged 12–13 years; higher transmission rates would be expected from younger children [25]. These findings highlight the importance of hygienic and public health measures after outbreaks or individual cases of cryptosporidiosis. Three of four confirmed secondary cases were primary caregivers, demonstrating not only that close contact is important for transmission, but also that adults should not consider themselves immune to such infections. Hand washing with soap should be the key message to all members of an affected household, especially for caregivers coming in direct contact with stools, soiled linen, bathwater or vomit from an infected person.

This is one of few studies to attempt to collect stool specimens from all household contacts after a cryptosporidiosis outbreak. Adding this to future studies will allow comparison of secondary transmission rates between different species and subtypes of *Cryptosporidium*. As *C. hominis* might be considered to be better adapted to the human ecosystem [26], it might have stronger potential for direct human-to-human transmission than *C. parvum*. It is possible that human asymptomatic secondary infections are more frequent and play a more important role in the epidemiology of *C. hominis* and in the proposed ‘anthroponotic’ IIc subtype of *C. parvum* [7] than in the epidemiology of the zoonotic IIa and IId *C. parvum* subtypes. Testing this hypothesis would require further studies of secondary household transmission from index cases of varying ages, combined with determination of species and subtypes.
